# Plasmodesmata Conductivity Regulation: A Mechanistic Model

**DOI:** 10.3390/plants8120595

**Published:** 2019-12-12

**Authors:** Yuri L. Dorokhov, Natalia M. Ershova, Ekaterina V. Sheshukova, Tatiana V. Komarova

**Affiliations:** 1 Vavilov Institute of General Genetics Russian Academy of Sciences, 119991 Moscow, Russia; ershovanatalie@gmail.com (N.M.E.); ekaterina.sheshukova@gmail.com (E.V.S.); t.komarova@belozersky.msu.ru (T.V.K.); 2 Belozersky Institute of Physico-Chemical Biology, Lomonosov Moscow State University, 119991 Moscow, Russia

**Keywords:** plasmodesmata (Pd), Pd-associated proteins, (1,3)-β-glucanase, callose synthase, class 1 reversibly glycosylated polypeptides, formin-like protein 1 and 2, pectin methylesterase, Pd located proteins, synaptotagmin

## Abstract

Plant cells form a multicellular symplast via cytoplasmic bridges called plasmodesmata (Pd) and the endoplasmic reticulum (ER) that crosses almost all plant tissues. The Pd proteome is mainly represented by secreted Pd-associated proteins (PdAPs), the repertoire of which quickly adapts to environmental conditions and responds to biotic and abiotic stresses. Although the important role of Pd in stress-induced reactions is universally recognized, the mechanisms of Pd control are still not fully understood. The negative role of callose in Pd permeability has been convincingly confirmed experimentally, yet the roles of cytoskeletal elements and many PdAPs remain unclear. Here, we discuss the contribution of each protein component to Pd control. Based on known data, we offer mechanistic models of mature leaf Pd regulation in response to stressful effects.

## 1. Introduction 

Due to their immobile lifestyle, plants are exposed to many abiotic and biotic stress factors [1]. A coordinated and generalized plant response to adverse environmental factors is accompanied by activation of both apoplastic and symplastic transport of macromolecules [2,3,4]. An understanding of the mechanisms of anti-stress reactions in plants has been facilitated by data obtained in recent years, indicating the role of intracellular traffic of secreted proteins with the participation of the endoplasmic reticulum (ER) and Golgi apparatus (GA) and the formation of an idea of alternative protein secretion pathways bypassing the GA [5,6,7,8]. Usually, with conventional secretion, secretory proteins with an N-terminal signal peptide are transported in secretory vesicles or secretory granules to the apoplast region via the ER, GA, and the subsequent endomembrane system. Passing through distinct GA compartments they undergo carbohydrate modifications and proteolytic processing [5,7,9,10,11]. However, recent plant secretome analysis has revealed considerable amount (up to 50%) of secreted proteins lacking a signal sequence [12]. These leaderless secretory proteins (LSPs) are not translocated into the ER and therefore cannot use the conventional protein secretion pathway with the participation of the GA. An alternative secretion route was termed as unconventional protein secretion (UPS) [6,7,13,14]. This definition also includes cases when plant proteins harbouring a signal peptide can traffic from this compartment to the extracellular space bypassing the GA [8,15]. Recent UPS models suggest that the ER is the central hub guiding proteins to an “external space” by circumventing the GA [8]. The extracellular space as well as the secretory route compartments’ lumen and the mitochondrial and plastid inner spaces are all considered as “external spaces” [7,8]. In contrast, proteomic studies have also revealed that the majority of LSPs are related to stress or pathogen infection [16], implicating the essence of the UPS pathway in dealing with various stress impacts [11]. Moreover, the increasing number of studies indicating a link between abiotic and biotic stress responses and secretion pathways offers research avenues towards understanding mechanisms involving the participation of plasmodesmata (Pd) in the plant stress tolerance [9,10,11]. With Pd, there is a direct connection between the symplast and the phloem that serves as a long-distance conductor in the vasculature, thus promoting rapid movement of materials between tissues both over short distances and over long distances throughout the plant. Pd provide a controlled symplastic exchange of both low molecular weight compounds and larger molecules, such as signaling molecules, transcription factors, non-cell-autonomous plant proteins [17,18], small regulatory RNAs and messenger RNAs [19,20], between plant cells, allowing general reactions in their growth processes and in response to stressful environmental factors [21,22,23]. Through Pd and phloem, photosynthesis products from the mesophyll of source leaves are exported to sink leaves and the plant growth point [24,25,26]. Sucrose, as a primary carbohydrate transported long distances in many plant species, is loaded into the phloem and unloaded into distal sink tissues. The productivity of agricultural plants largely depends on the efficiency of sucrose and other carbohydrates export from the mesophyll to the phloem through Pd, and further to the ripening fruits and grains [17,27,28,29,30,31,32,33,34].

The important role of Pd in stress response is universally recognized [21,22,23]. At the same time, the mechanisms for regulating Pd functions and permeability after stress impacts are still not fully understood. If the negative role of callose in the control of intercellular molecular flow via Pd is convincingly confirmed experimentally [35,36,37], the role of cytoskeletal elements and protein components, called Pd-associated proteins (PdAPs), is mostly unclear [32,38]. The analysis of the Pd proteome revealed that most PdAPs are secreted proteins [39] that might be involved in controlling Pd permeability under abiotic and biotic stress. 

In this review, we analysed the characteristics of intracellular PdAP trafficking and assessed the contribution of each protein component to controlling Pd. A mechanistic view on Pd aperture regulation via callose and secreted proteins in plants is also presented. 

## 2. Substructural Architecture of Plasmodesmata

**Figure 1:**
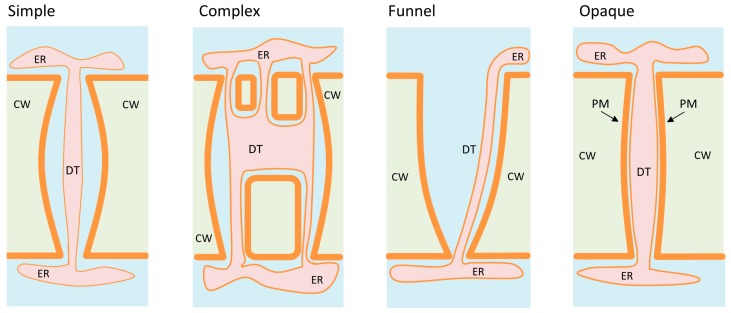
Different types of Pd. The Pd vary from simple with one channel connecting the adjacent cells to complex Pd with several channels that could merge. Also, there are some specific types of Pd such as funnel-shaped ones which are characteristic of the root tissues and opaque Pd found in the mitotically active tissue in the cell walls after cytokinesis. PM, plasma membrane (bold orange line); CW, cell wall (pale green); DT, desmotubule; ER, endoplasmic reticulum.

The structure of a typical simple Pd (Figure 1), whose length is approximately 100 nm with a diameter of 30 nm, is only clearly distinguishable by transmission electron microscopy [40]. In addition to the plasma membrane (PM) lining the inner surface of holes in the cell wall, Pd include a centrally located desmotubule (DT) which is derived from the compaction of ER membranes [41]. The cytoplasmic sleeve between the DT and PM defines the space available for intercellular trafficking and is filled with the combined cytoplasm of neighbouring cells (Figure 1). 

Primary Pd in plants are formed at the end of cell division when the ER penetrates into the developing cell plate, creating a cytoplasmic nanopore [42]. However, depending on the nature of the tissue and its age, the structure and configuration of Pd vary. Beyond Pd with a single nanochannel, referred to as simple Pd, there are also more complex Pd with double or even multiple branched nanochannels [40] (Figure 1). 

Another form of Pd called a “funnel Pd” is found in *Arabidopsis* roots between protophloem sieve element (PSE) cells and phloem-pole pericycle (PPP) cells [43]. The location of the wide aperture of the “funnel Pd” inside the PSE is thought to be more effective than a simple Pd configuration for unloading dissolved substances in PPP (Figure 1).

Finally, in mitotically active tissue in the cell walls after cytokinesis, in addition to simple Pd, it is possible to discern Pd called “opaque”, in which there is such a tight contact between the ER and PM that there is no visible intermembrane space (Figure 1). However, in the absence of a visible sleeve in “opaque” Pd, intercellular movement of macromolecules such as GFP is still allowed [44].

Pd permeability is determined by the size of the largest molecule that can pass through it, creating a criterion termed the size exclusion limit (SEL) which is based on the dimensions of the folded molecule (or Stokes radius). Pd permeability testing methods have been evaluated in detail recently [32]. Branched secondary Pd have a decreased SEL compared with the primary Pd, restricting the movement of large molecules [45]. It is believed that the transition from immature to mature tissues correlates with increased Pd branching and a restriction in transport ability. The Pd SEL can be modulated in response to biotic/abiotic stresses, but Pd symplastic connectivity is strongly regulated in the space and over time [17,22,32,46,47].

## 3. Plasmodesmata-Associated Proteins (PdAPs)

The analysis of the current data enabled us to select PdAPs involved in the regulation of Pd permeability (Table 1). PdAPs can be divided into the following two groups: non-secreted cytoplasmic proteins including ER-resident proteins and secreted proteins. 

### 3.1. Non-Secretory Pd Proteins

**Figure 2:**
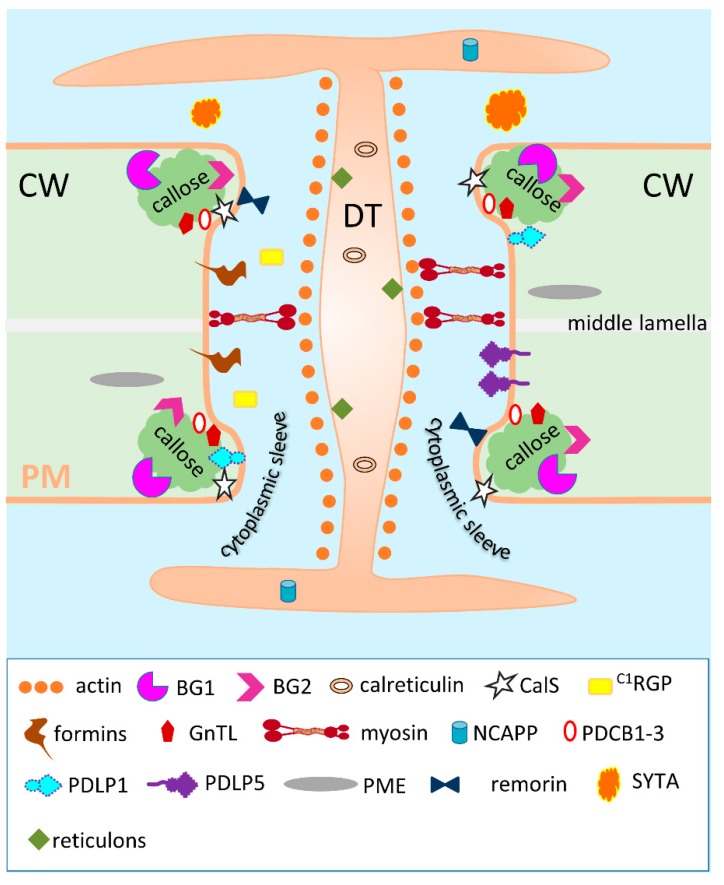
The structure of a typical simple Pd. Plasmodesmata is a pore in the cell wall (CW) lined with a plasma membrane (PM). It contains a centrally located desmotubule (DT) which originates from the endoplasmic reticulum of the adjacent cells. The space between the PM and the DT, a cytoplasmic sleeve, is available for the intercellular trafficking. Molecules of actin and myosin are resident Pd proteins as they are components of the cell cytoskeleton that extends from cell to cell through Pd and is involved in regulating Pd permeability. Callose (designated with a green cloud-like shape) is a linear β-1,3-glucan molecule and it is considered as a key plasmodesmal marker that controls Pd permeability. BG1 and 2, (1,3)-β-glucanase 1 and 2, callose-degrading enzymes; calreticulin, Ca^2+^-sequestering protein chaperone; CalS, callose synthase; ^C1^RGP, Class 1 reversibly glycosylated polypeptide; formins, proteins involved in actin stabilization and anchoring cytoskeletal structures to membranes; GnTL, β-1,6-N-acetylglucosaminyl transferase-like enzyme; NCAPP, non-cell autonomous pathway protein; PDCB1-3, Pd callose binding proteins 1-3; PDLP1 and 5, Pd-located protein 1 and 5; PME, pectin methylesterase, an enzyme that performs de-methyl esterification of pectin; remorin, a protein associated with PM raft-like structures; SYTA, *A. thaliana* synaptotagmin which is a tethering protein that maintains ER morphology and stabilizes the formation of ER–PM contacts; reticulons, ER-tubulating proteins participating in the formation of the Pd DT.

This group of proteins include non-secreted cytoplasmic proteins found in the Pd space as well as proteins associated with the DT and Pd plasma membrane (Figure 2).

#### 3.1.1. Actin, Myosin and Tubulin

The cytoskeletal proteins actin and myosin have been implicated with the structure and function of Pd (Figure 2). The presence of these proteins in cytoplasmic sleeves in the Pd cavity was discovered long ago using immunodetection method [48,49,50]. Compared with actin and myosin, the role of tubulin in the functioning of Pd is not well understood. There are only speculations about tubulin’s participation in long distance transport [48,86].

#### 3.1.2. Synaptotagmins

Analysis of the *Arabidopsis* Pd proteome revealed several synaptotagmins (SYTA) [39]. In addition to endosome recycling, AtSYTA participates in plant viral movement protein (MP)-mediated trafficking of begomovirus Cabbage leaf curl virus (CaLCuV) and Tobacco mosaic virus (TMV) genomes through Pd. In AtSYTA knockdown *Arabidopsis* lines, CaLCuV systemic infection was delayed, and cell-to-cell spread of TMV and CaLCuV movement proteins was inhibited [60].

The mechanism of AtSYTA participation in tobamovirus cell-to-cell movement was clarified by a study by Levy et al. [59], which showed that tobamovirus Turnip vein-clearing virus (TVCV) MP was able to (i) interact with SYTA, (ii) remodel ER-PM contact sites and (iii) form virus replication sites for further cell-to-cell movement. It has been suggested that TVCV MP-mediated recruitment of SYTA leads to its accumulation in the Pd cavity [59]. Most likely, such recruitment begins with the binding of the 50-aa TMV MP Pd-localization sequence [87,88] with SYTA, as was recently shown [62]. This event can be considered as the beginning of the process, leading to the dilatation of Pd. According to the hypothetical mechanism proposed by Pitzalis & Heinlein, MPs interact with one SYTA form, SYT1, the length of which, as the tethering molecule, is controlled through interaction with myosin VIII. The model assumes that the chain of events triggered by the interaction of MP and SYT1 leads to compression of the DT lumen, an increase in the size of the cytoplasmic channel and, accordingly, Pd SEL [89].

#### 3.1.3. Remorin

Remorin is another non-secreted cytoplasmic protein lacking a signal sequence. Remorin is extremely hydrophilic, has a proline-rich N-terminus and is synthesized in the cytosol and targeted to the cytoplasmic surface of the Pd PM [90]. The C-terminal part of the protein was predicted to be a coiled-coil, suggesting that the protein interacts with other macromolecules. The localization of remorin was studied using immunogold labelling coupled with electron microscopy, which allowed its visualization not only in plant PM domains but also at the Pd cavity [56]. Remorin participation in virus cell-to-cell traffic was confirmed by data indicating the following: (1) its ability to interact physically with the Potato virus X (PVX) triple gene block protein 1 and (2) its expression levels negatively correlate with the cell-to-cell movement of PVX [56].

#### 3.1.4. Calreticulin

Calreticulin, a Ca^2+^-sequestering protein chaperone responsible for the ER unfolded protein response and highly conserved between different species, also accumulates within the Pd in plants [51,91,92,93,94]. In general, calreticulin binds to misfolded ER luminal proteins and prevents them from being exported from the ER to the GA, functioning as a quality-control chaperone. *Arabidopsis* calreticulin-1 (AtCRT1) is 425 aa in length with 22-aa signal sequence mandatory for Pd localization [95], three N-glycosylation sites and the C-terminal retention sequence HDEL that prevents its exit from the ER lumen. AtCRT1 binds TMV MP *in vitro* and *in vivo* and colocalizes with this protein in Pd [52]. If, in response to stressful effects, CRT accumulates in the ER lumen in the area of the DT in the Pd central cavity, it can not only block the movement of molecules along Pd sleeves but also prevent the appearance of TMV MP in Pd. This assumption is supported by experiments by Chen et al., which showed that overexpression of AtCRT1 hinders TMV cell-to-cell movement and blocks MP accumulation in the Pd [52]. As an alternative explanation, it is possible that the presence of large quantities of AtCRT1 within plant cells redirects TMV MP from Pd to the microtubular network [52].

#### 3.1.5. Non-Cell-Autonomous Pathway Proteins (NCAPPs)

NtNCAPP, which is found in tobacco [54] in two highly homologous forms, NCAPP1 and NCAPP2, has high homology to aldose 1-epimerase-like protein and all the features of a protein secreted by the GA, including a 24-aa signal sequence and predicted N-glycosylation sites. Immunogold labelling analysis showed the presence of NCAPP1 in cells near the Pd orifice but not in the central cavity. This PdAP was identified as an interaction partner of the 16-kD *Cucurbita maxima* (pumpkin) phloem non-cell autonomous protein CmPP16 which moves from cell to cell and participates in the RNAs intercellular transport [96]. In experiments using a dominant-negative mutant NCAPP1^Δ1–22^, its important role in the intercellular transport of CmPP16 and TMV MP was shown. A model [97] has been proposed where NCAPP1 promotes the translocation of some NCAPs through Pd after their reciprocal phosphorylation and glycosylation [98]. 

#### 3.1.6. Reticulons 

Reticulons (RTNLB), as family of membrane ER-tubulating proteins play a role in the formation of the Pd DT, which regulates the movement of small molecules via cytoplasmic sleeves [57,99,100]. Of the seven *Arabidopsis* RTNLBs, Pd essentially enriched only with RTNLB3 and RTNLB6. Although all AtRTNLBs lack an ER signal peptide, their translocation into the ER is likely directed by internal transmembrane domains [101]. It is unclear whether the GA is involved in the secretion of *Arabidopsis* reticulons, but the related human RTN3 uses it [102]. The results from studies of AtRTNLB3 and AtRTNLB6 showed high degree of colocalization of these proteins with TMV MP in the Pd central cavity [57]. It has been suggested that RTNLBs from the Pd central cavity could promote a mechanism for reducing Pd permeability through the expansion of the DT while viral MPs provide Pd gating by disrupting the DT scaffold and perturbing protein-lipid or protein-protein interactions.

### 3.2. Secreted PdAPs 

This category includes PdAPs that obey the principles of traditional or conventional secretion, regardless of their final localization in Pd (Figure 2).

#### 3.2.1. Callose-Degrading β-1,3-glucanases (BG)

The callose contents at Pd depends on the activity of (1,3)-β-glucanases (BGs). Based upon the protein domain structure and sequence, *Arabidopsis* BGs can be classified into five groups with at least 50 members [63]. BGs contain an N-terminal secretion signal. Some members contain a hydrophobic C-terminal sequence that includes a predicted glycosylphosphatidylinositol (GPI)-anchor attachment motif for targeting the protein to the cell membrane [73] and to Pd [85]. Callose degradation at the *A. thaliana* Pd depends on the activity of three GPI-anchored BG proteins, including β-1,3-glucanase_putative Pd-associated protein (AtBG_ppap), plasmodesmal-localized β-1,3-glucanase 1 (PdBG1), and plasmodesmal-localized β-1,3-glucanase 2 (PdBG2) [35,73]. PdBG3 is also localized at Pd, but its role in callose degradation is not proven [73].

*Arabidopsis* BGs are conventionally secreted proteins associated with PM and Pd. However, in detailed analysis using the plasmolysis method, AtBG_ppap, for example, could be seen to recede from the cell wall along with PM [35]. Thomas et al. suggest that unlike PDLP1a, β-1,3-glucanase moves to Pd via the PM and maintains only a superficial physical association with Pd [78].

Biotic and abiotic stress have significant impacts that affect the expression and localization of the BGs. Most BGs, including AtBG2 and AtBG3, are regulated at the transcriptional level during stress [65]. For example, while TVCV did not affect *AtBG_ppap* transcription, the transcription of an AtBG2-encoding gene was enhanced [64]. Analysis also revealed that AtBG2 is present in the extracellular space and colocalizes with callose near Pd orifices in healthy cells, while its secretion ceases and it associates with viral MP in Pd in the TMV-infected cells [64]. 

In general, the link between BGs activity and viral infection has been noted for a long time [36]; even in 1972, Moore and Stone proposed hypothesis that “the fact that it can depolymerize callose suggests that it could have a role in facilitating the spread of virus through removal of callose deposits or by modification of cell wall structures” [103]. This hypothesis was confirmed in experiments with TMV-infected plants treated with methanol [104]. It has been shown that genes encoding BGs are sensitive to methanol released from wounded plants. In contrast, both methanol treatment and the introduction of a gene encoding BG into the cell resulted in the increased gating capacity of Pd, which created favourable conditions for viral cell-to-cell movement and ultimately led to increased reproduction of TMV in the plant. 

#### 3.2.2. Pd-Associated Callose Binding Proteins (PDCBs)

PDCBs are secreted proteins that appear in the extracellular space through participation of the GA. *Arabidopsis* has 11 PDCB-like proteins, including PDCB2 and PDCB3, which are the most studied [74]. All PDCBs comprise an N-terminal signal peptide, an X8 domain (CBM43), an unstructured region in the middle and a GPI-anchor motif at the C-terminus. The *At5g61130* gene encodes a GPI-anchored PDCB1 protein with N- and C-terminal domains that are cleaved during processing from the preprotein. Mature PDCB1 has a length of 153 amino acids, two potential N-glycosylation sites and a GPI-anchor-amidated Ser172. It was determined that PDCB1 was located at the neck region of Pd where callose becomes deposited [74]. It was also established that PDCB1 binds to callose through its X8 domain. PDCB1 does not have callose-synthesis enzymatic activity; however, its overexpression, for unclear reasons, leads to an increase in callose accumulation, and decreased Pd permeability in a GFP-movement assay [74].

#### 3.2.3. Plasmodesmata-Located Protein 1 (PDLP1)

PDLP1 was the first identified representative of the 8-protein family in *Arabidopsis*. These proteins are type I membrane proteins with a molecular mass ranging from 30.2 to 35.3 kDa and comprising an N-terminal signal peptide, a large region containing two similar domains annotated as domains of unknown function 26 (DUF26), a single transmembrane domain (TMD), and a short C-terminal tail. DUF26 domains have a conserved C-X8-CX2-C motif, which is distinct from the Cys-rich regions found in S-locus glycoproteins [78]. 

Studies of PDLP1, as the first representative of a new family of proteins, confirmed that it uses a traditional secretion mechanism involving the GA, which was sensitive to brefeldin A and showed sensitivity to disruption of COPII-mediated ER-export by the GTP-locked form of Sar1. Expression in transgenic *Arabidopsis* showed that PDLP1 fused with GFP was located as punctuated spots on the cell wall and that the fluorescence persisted on the cell wall after plasmolysis. Further evidence that these punctuate sites were Pd was obtained by colocalization methods with callose and TMV MP [78]. Functional tests confirmed that PDLP1 can regulate intercellular transport by affecting Pd. PDLP1 deletion analysis has shown that its single TMD is required for targeting to Pd. PDLP1 acts as a negative Pd regulator because its overexpression causes restricted cell-to-cell trafficking due to callose deposition [78]. PDLP1 also promotes the transport of viruses that use tubule-guided movement by redundantly interacting with tubule-forming Grapevine fanleaf virus [75] and Cowpea mosaic virus (CPMV) [105] MPs within Pd. As suggested, viruses can subvert the functions of PDLPs proteins to facilitate the cell-to-cell movement of entire virions [22].

#### 3.2.4. Plasmodesmata-Located Protein 5 (PDLP5)

PDLP5 is another representative of the 8-member family of *Arabidopsis* PDLPs. Although it has only 30% amino acid sequence identity to PDLP1, it has all the features of secreted proteins of this family and is a type I transmembrane protein containing a cysteine-rich extracellular domain and a transmembrane domain with a short C-terminal tail [79,81]. For intracellular movement, PDLP5 uses the GA and it has potential N-glycosylation sites [80]. Similar to PDLP1, PDLP5 acts as a negative Pd regulator that suppresses Pd trafficking and confers enhanced innate immunity against bacterial pathogens in a salicylic acid–dependent manner [31]. For this topic, it is important to note the differences between PDLP1 and PDLP5 in Pd localization. If PDLP5 is localized to the Pd membranes, then PDLP1 is distributed both throughout the Pd membrane [106] and along the PM [107]. Recently, a possible cause for the differences in the behaviour and localization of PDLP1 and PDLP5 has been identified [82]. Pd-lining plasma membrane is known to contain specific regions in which sphingolipids interact with sterols to create specialized domains [106,108,109], where GPI-anchored proteins accumulate [39,56]. Moreover, sphingolipid biosynthesis is very important for modulating Pd function [110]. Thus, the loss of the function of the PHLOEM UNLOADING MODULATOR (PLM) gene, which encodes the protein involved in the synthesis of sphingolipids, leads to a change in the function and structure of the Pd – specifically, the disappearance of cytoplasmic sleeves in the Pd [110]. Ning-Jing et al [82] revealed that Pd membranes are enriched with specific t18:0-based sphingolipids, compared with these sphingolipid levels in the plasma membrane. In examining the *Arabidopsis* sld1 sld2 double mutant, which lacks sphingolipid long-chain base 8 desaturases 1 and 2, leading to the accumulation of t18:0-based sphingolipids in the Pd membranes, the authors found increased accumulation of PDLP5, which has specific binding affinity for phytosphinganine (t18:0), in leaf epidermal cells. The double mutants also showed increased callose accumulation, decreased Pd permeability and enhanced resistance to the fungal-wilt pathogen *Verticillium dahlia* and the bacterium *Pseudomonas syringae* pv tomato DC3000. Ning-Jing et al [82] attributed the specific binding of PDLP5 to t18:0-based sphingolipids by the existence of the sphingolipid-binding motif TXXILXXVF [111] in the PDLP5 TMD (LAIIIGIVTLIILLVVFLAFV). It is important to note that this motif is absent in the PDLP1 TMD (IALAVGGVFVLGFVIVCLLVL), which explains the difference in the localization of these proteins.

PDLP5 was found to colocalize with TMV MP at the central region of Pd channels. Quantitative analysis of immunogold staining for TMV MP and PDLP5 showed that the labelling was highly specific and convincingly confirm their association with Pd. In accordance with the function of a negative Pd regulator, ectopic expression of PDLP5 delayed the systemic movement of TMV-GFP in *N. benthamiana*. In contrast, downregulation of PDLP5 alone was sufficient to enhance Pd permeability [79]. By contrast, ectopic expression of PDLP5 in *N. benthamiana* did not affect the systemic movement of *Cucumber mosaic virus* (CMV), which may indicate a difference in the functioning mechanisms of TMV and CMV MPs [22]. In turn, Turnip crinkle virus (TCV) can also overcome PDLP5-mediated Pd constriction in *A. thaliana* plants [77]. Thus, plants with PDLP5 overexpression can be a differential test system to identify the differences in the mechanisms of virus cell-to-cell movement.

**Figure 3:**
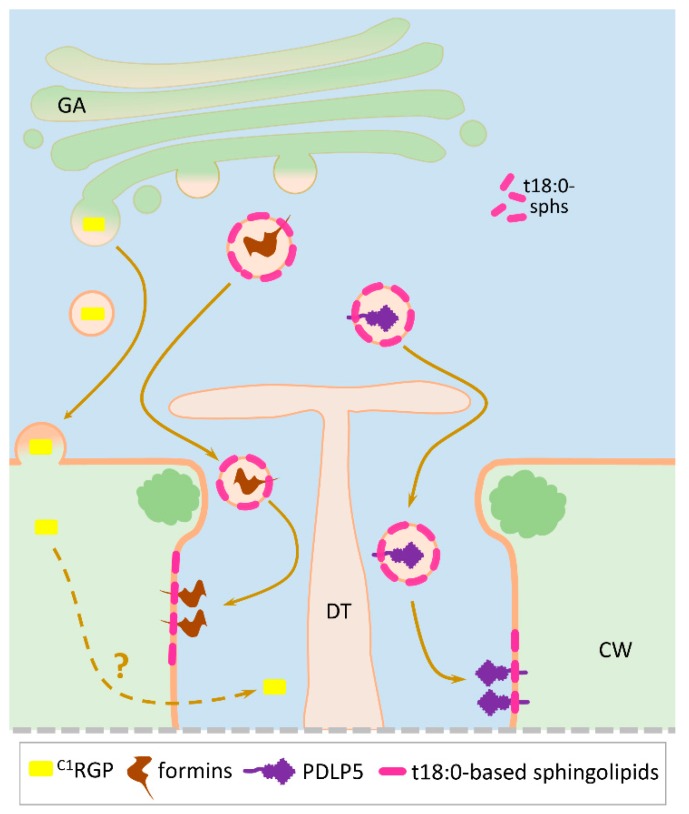
Hypothetical model showing the targeting of the secreted PdAPs into the cytoplasmic sleeve. Schematic representation of PDLP5 vesicular transport involving specific t18:0-based sphingolipid traffic with the final destination in the Pd cavity. Formins are hypothesized to be delivered to the Pd in a similar way. ^C1^RGP is secreted to the cell wall and reaches its location in the Pd cytoplasmic sleeve by an unknown mechanism. Sphingolipids are designated as purple rods. CW, cell wall; ^C1^RGP, Class 1 reversibly glycosylated polypeptide; DT, desmotubule; GA, the Golgi apparatus; PDLP5, Pd-located protein 5.

It is agreed [79] that the identical localization of PDLP5 and TMV MP at the central region of Pd channels raises questions and requires additional research. The data on the preferential localization of specific t18:0-based sphingolipids and the associated with them PDLP5 in the Pd membrane, as described above, support the idea of co-localization of PDLP5 and TMV MP. It is possible that not only callose deposition but also competition between PDLP5 and TMV MP for binding to common Pd factors are critical for Pd regulation. If PDLP5 and TMV MP indeed occupy the same Pd subdomain, this means that, contrary to the transmembrane nature of the secretory protein, PDLP5 should be at the same site as TMV MP, which is localized on the cytoplasmic surface of the ER [112]. In accordance with our hypothesis, this contradiction could be resolved through a mechanism involving specific t18:0-based sphingolipid traffic, that results in PDLP5 localization in the Pd cytoplasmic sleeve (Figure 3).

#### 3.2.5. β-1,6-N-acetylglucosaminyl Transferase-Like Enzyme (GnTL)

In the selection of proteins that bind to Pd-localized calreticulin, Zalepa-King et al. isolated β-1,6-N-acetylglucosaminyl transferase-like enzyme (GnTL) among other Pd-associated proteins in *A. thaliana* [72]. AtGnTL is 346-residues protein containing an amino-terminal signal peptide followed by a catalytic domain (GnT) characteristic of the glycosyltransferase protein family, which catalyses the transfer of a specific activated sugar moiety from a donor molecule to an acceptor via a glycosidic bond in the GA. Subcellular localization studies using confocal microscopy observed AtGnTL at Pd within living plant cells. AtGnTL was confirmed as a Pd-located protein in a plasmolysis test. AtGnTL-mCherry retained its punctate localization pattern within the cell wall. The authors suggest that AtGnTL and TMV MP are likely to have different sites in Pd that are available for binding to calreticulin [72]. Although the authors did not directly show that AtGnTL expression suppresses Pd permeability in this work, it can be assumed that AtGnTL is able to function as a negative regulator of Pd given its association with calreticulin, the expression of which blocks TMV infection [52].

#### 3.2.6. Cell Wall Pectin and Pectin Methylesterase (PME) as Factors Controlling Pd Permeability

Primary Pd are grouped in clusters called pit fields [113]. The cell wall that surrounds Pd is also an important component of its structure [83]. It is known that the cell wall material directly around the Pd is rich in pectin and depleted in cellulose. It is believed that the cell wall enriched in pectin is more flexible than cellulose fibres, and this may allow dynamic changes in the Pd structure and functions that occur in response to a number of environmental signals [29,44,83]. This view has been confirmed by known data about the stress-induced enzyme, PME, involved in pectic homogalacturonan (HG) de-esterification *in muro* [114], showing its accumulation in the cell wall near Pd [84].

An important consequence of pectin demethylation that affects Pd permeability is the stimulation of multiple methanol-inducible genes (MIGs) expression [104]. Some MIGs are involved in defense reactions, while others affect cell-to-cell trafficking. Tobacco *BG* coding for the related to basic vacuolar BG isoform, a previously unidentified gene (*MIG-21*), and *NCAPP* increased the Pd gating capacity, which in turn stimulated TMV cell-to-cell movement [104]. It is known that PME interacts with TMV MP *in vitro* [115,116], suggesting that PME may be directly involved in the cell-to-cell movement of plant viruses [117].

#### 3.2.7. Callose Synthase (CalS)

Callose synthase (CalS) is an enzyme involved in the metabolism of callose. *A. thaliana* AtCalSs are encoded by 12 glucan-synthase-like genes (*AtGSL1* to *AtGSL12*) and are large proteins (1770–1950 amino acids) possessing a large central catalytic domain which includes a UDP-glucose catalytic site and a glycosyltransferase domain surrounded by multiple transmembrane domains [69,118]. CalSs are located in the PM and exhibit high substrate specificity for the nucleotide sugar uridine diphosphate glucose (UDP-glucose) [37,47,119]. Several isoforms of AtCalSs were found in the proteome of *trans*-Golgi-network-derived vesicles [120] indicating that these proteins are conventionally secreted. The final step of their secretion and fusion to the PM involves EXOCYST subunit EXO70 family proteins [121]. CalS performs its function in conjunction with UDP-glucose transferase 1 (UGT1), Rho-like GTPase (Rop), RabA4c, tubulin, phragmoplastin (Phr), sucrose synthase (SuSy), and annexin (ANN), thus forming a highly specialized protein complex for callose biosynthesis [122]. 

CalSs are capable of post-translational regulation. Thus, in response to various abiotic and biotic stresses, some *Arabidopsis* CalSs are phosphorylated, which affects their activity [122]. Since callose is involved in the control of Pd, it can be assumed that CalSs are also located in Pd [47]. Indeed, a study of mutant *Arabidopsis gsl12/cals3* lines confirmed this assumption [123]. It has been shown that the GFP-CalS3 fusion protein can be localized in Pd in transfected leaves. Moreover, gain-of-function mutations in CalS3 resulted in increased accumulation of callose at the Pd, a decrease in the Pd aperture and reduced intercellular trafficking [123].

#### 3.2.8. Formins

Plant formins are evolutionarily conserved eukaryotic proteins involved in actin nucleation and cytoskeletal organization. Typical plant Class I formins are integral membrane proteins that can anchor cytoskeletal structures to membranes. The *Arabidopsis* Class I formin AtFH1 accumulates in actin-rich regions of the cortical cytoplasm and associates with Pd [71]. The *Arabidopsis* Class II formin AtFH2 was also detected in Pd and is suggested to regulate Pd permeability by anchoring actin filaments to Pd [70]. Formins are secreted proteins that have a signal sequence and use the GA for intracellular movement. AtFH2 is 894 amino acids in length with 20-aa signal sequence and multiple N-glycosylation sites. Similar to PDLP1 and PDLP5, AtFH1 and AtFH2 also have 21 amino acid transmembrane domains. Moreover, AtFH2 TMD, in contrast to AtFH1, contains a 9 amino acid region very similar to the sphingolipid-binding motif. Despite having all the properties of a secreted protein, AtFH2 appears to interact with actin microfilaments in the Pd cavity. Moreover, AtFH2 caps and stabilizes actin filaments, thereby regulating cell-to-cell trafficking by tethering actin filaments to the membrane at Pd. Since the polymerization of actin filaments reduces the clearance of Pd, AtFH2-mediated stabilization of actin filaments can play a negative role in Pd permeability. Indeed, loss of function of AtFH2 increased Pd SEL. These mutant plants, which have more permeable Pd, became more susceptible to a Cucumber mosaic virus [70]. Thus, plant formins participate in the Pd closing mechanism.

#### 3.2.9. Class 1 Reversibly Glycosylated Polypeptide (^C1^RGP) 

^C1^RGP is a member of the reversibly glycosylated polypeptides family that also includes ^C2^RGP [124], which self-glycosylates in the presence of certain nucleotide sugars [125,126,127]. Analysis of At^C1^RGP showed that it is a secreted protein. Despite having no signal sequence, this protein is found in the GA and, ultimately, in the Pd [67]. In plasmolysed cells, the association of At^C1^RGP fused with GFP (At^C1^RGP-GFP) with the cell wall remains unchanged. The hypothesis that At^C1^RGP is delivered to Pd via the GA was confirmed by experiments where brefeldin A which prevented the appearance of At^C1^RGP-GFP in Pd [67]. To explain the mechanism of association of ^C1^RGP with Pd, Sagi et al. suggested that “upon fusion of the GA to the PM of a Pd or in the vicinity of a Pd, ^C1^RGPs would be attached to the PM facing the cytoplasmic sleeve of the Pd. The integration of ^C1^RGPs onto the cytosolic facing side of the plasmodesmal PM could be a factor in establishing the size exclusion limit for soluble proteins” [67]. Subsequently, this hypothesis was confirmed by experiments showing changes in the expression levels of the gene encoding At^C1^RGP. It has been shown that *At^C1^RGP* overexpression leads to a reduction in Pd permeability for photoassimilates and TMV cell-to-cell movement [68]; in contrast, reduced levels of At^C1^RGP increase intercellular transport via Pd, and TMV exhibited accelerated systemic spread [66]. 

It is important to note here that when ^C1^RGP was added to a class of secreted PdAPs (Table 1, Figure 2 and Figure 3), we proceeded from the data obtained by studying the ectopic expression of At^C1^RGP in tobacco [67]. It is important to note that studies of the Rautengarten et al group [128] showed that At^C1^RGP1 and At^C1^RGP2 are cytosolic Golgi-associated enzymes belonging to the UDP-L-arabinopyranose (UDP-L-Ara) mutases, which catalyse the formation of UDP-Araf from UDP-Arap. In addition, the authors reported (results not shown) that they “did not observe any fluorescence originating from plasmodesmata, consistent with previous studies in *Arabidopsis*” [125,127,129]. This contradiction is apparently a consequence of the differences in the secretion mechanisms of tobacco and *Arabidopsis*; in our studies, the *Nicotiana benthamiana* RGP (Nb^C1^RGP), which has 90% homology with At^C1^RGP, was found in the Pd.

## 4. Mechanisms of PdAP Participation in Intercellular Cytoplasmic Connectivity

Plant intercellular cytoplasmic connectivity can be regulated by several mechanisms [17,27,28,31,34]. The first mode of regulating symplastic transport can be carried out by changing the density of primary Pd per unit area of the cell wall. As cells grow and the surface of their cell walls expand, the density of primary Pd is decreased. 

The second way to change the intercellular conductivity is the modification of Pd structure when a transition from primary to secondary Pd occurs. The study of the sink-source transition in tobacco leaves has shown how this mechanism works [28] when a large number of simple primary Pd are lost and the contents of branched secondary Pd increase [26,40]. 

The third mechanism involves a change in Pd permeability based on the function of its structural elements and PdAPs. This mode is especially important for secondary Pd from mature leaves during rapid reactions to stress [21,22,23]. This mechanism includes mechano-sensing Pd closure in response to tissue damage or osmotic shock, and has recently been reviewed [130]. It is believed that intracellular pressure forces acting on the dumbbell-shaped ER DT cause the displacement of this complex, leading to Pd closure.

Although a relatively large number of potential Pd components have been identified [31,32,33,38], the current understanding of the mechanisms for regulating the Pd conductivity is based on the participation of callose, which was revealed by electron microscopy studies near and within the Pd channel [131]. Our analysis of PdAPs (Table 1) shows that features of secreted proteins also play an important role in the functioning of Pd. We assume that the impact of ER-GA secretion in the function of Pd-associated proteins increases under stress when the plant needs to provide sink leaves and a growth point with carbohydrates synthesized in mesophyll from source leaves. In general, all the PdAPs presented in Table 1 can be conditionally divided into two unequal groups. The first group includes PdAPs, which can be considered as negative Pd regulators and includes actin, remorin, PDLP1 and 5, GnTL, ^C1^RGP, formins, reticulons, calreticulin. The small second group includes positive Pd regulators such as myosin, SYTA, BGs, PME and NCAPP. Many viral MPs can be also included in this group [132].

**Figure 4:**
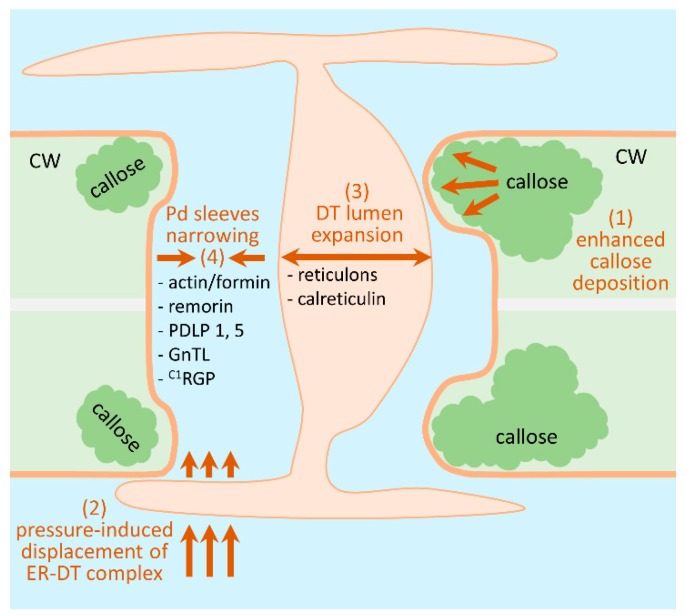
Schematic representation of plasmodesmata permeability negative regulation. (**1**) callose depositions around Pd neck; (**2**) stress-induced increasing of the intracellular pressure leading to the compression of the ER DT and blocking Pd entrance; (**3**) ER DT lumen expansion as a result of calreticulin and reticulons deposition; (**4**) narrowing of the cytoplasmic sleeves.

If we proceed from the Pd structure (Figure 1 and Figure 2) along channels in the cell wall that contain the dumbbell-shaped ER DT inside them and ducts, or Pd sleeves, surrounding DT, then there are several ways to block the movement of molecules through these structures (Figure 4).

First, is stimulating callose deposition around Pd [36,37]. Second, by increasing the intracellular pressure, thereby compressing the ER DT and blocking the entrance to Pd in accordance with the model proposed by Park et al. [130]. Third, the deposition of calreticulin and reticulons in the DT lumen suggested by Knox et al. leads to the expansion of the ER DT lumen [57]. Fourth, narrowing the Pd sleeves, as probably occurs through the participation of the actin-formin complex [70], as well as the accumulation of remorin [56], PDLP1 and 5 [75,76,77,78,79], GnTL [72], and ^C1^RGP [66,67,68]. Pd opening, as a process opposite to closing, can include (1) the removal of callose and (2) the displacement of negative regulators from the Pd structure. PME and NCAPP can also have an indirect positive effect on Pd dilation. Since most PdAPs are secreted proteins, modification of the intracellular protein trafficking pathways is also involved in Pd control. 

## 5. Conclusions and Outlook 

Although the important role of Pd in the development of tissues and plant organs is universally recognized, the mechanisms for regulating Pd functions after stress impact are still not fully understood. The reactions of mature leaves Pd to stress are controversial due to the nature of the stressful agents, and, most importantly, to the different time points after stress impact. It is generally accepted that stress responses include Pd closure as a result of reversible callose deposition.

Our analysis indicates a variety of protein factors involved in generalized Pd-mediated stress responses. It is still unclear which genes and protein factors encoded by stress-induced genes are activated in source leaf cells to provide a fast and generalized stress response, leading to an increase in Pd conductivity and the outflow of photoassimilates to sink leaves. We assumed that the search for these genes should be carried out among genes with different levels of mRNA accumulation in leaves and roots as well as after stress challenge. The analysis of the PdAPs presented in Table 1 allows to evaluate the role of secreted proteins in the functioning of Pd. The proteomic study of a Pd-enriched *Arabidopsis* cell wall fraction showed that most PdAPs are secreted proteins. The Pd structure, including its extracellular and endocellular parts, involves both cytoplasmic non-secreted proteins and secreted proteins control, such as in the case of callose metabolism. It was previously believed that the list of cytoplasmic PdAPs located directly in the intracellular cytoplasmic sleeve was limited by actin and myosin. However, PdAPs that have all the properties of proteins secreted by the GA have recently been revealed, though their final localization is in the Pd intracellular space. With the recent discovery of the specific role of t18:0-based sphingolipids in PDLP5 localization in the Pd cytoplasmatic sleeve this mechanism may also be suggested for formins (Figure 3).

The putative mechanisms for the participation of PdAPs in the regulation of Pd presented in Figure 4 are diverse, but ultimately come down to the means of closing/opening the molecule passage through Pd sleeves surrounding the DT. 

## Figures and Tables

**Table 1:** Pd-associated proteins (PdAPs) involved in regulation of Pd permeability.

Protein	Function/Description	Signal Sequence	Predicted N-Glycosylation Sites	Pd Localization	Protein Relocation After Stress Impact	References
Pd non-secretory proteins						
Actin, myosin and tubulin	Actin-myosin filaments and tubulin are localized within the Pd cytoplasmic sleeve and negatively control Pd permeability	No	No	Cytoplasmic sleeves in the Pd cavity	No	[48,49,50]
*A. thaliana* calreticulin-1 (AtCRT1) (UniProt O04151)	Ca^2+^-sequestering protein chaperone and ER protein that negatively control Pd permeability	Yes	Asn59, Asn154, Asn399	Associates with the desmotubule	Yes	[51,52,53]
Tobacco non-cell autonomous pathway protein (NCAPP) (UniProt Q947H5)	NCAPP has homology to aldose 1-epimerase; positively controls Pd permeability and PME/methanol production	Yes	Asn76 and Asn100	Localizes in the ER	N/A	[54,55]
Remorin	Associates with PM raft-like structures and probably serves as a negative regulator of Pd permeability. Antagonist of Potato virus X triple gene block protein 1	No	No	In the cytosolic surface of the Pd plasma membrane	N/A	[56]
*A. thaliana* reticulons, AtRTNLB3 (UniProt Q9SH59) and AtRTNLB6 (UniProt Q6DBN4)	ER-localized proteins with three TM domains that negatively control Pd permeability	No	No	Accumulates in the desmotubule	N/A	[57,58]
*Arabidopsis* synaptotagmin SYTA (AtSYTA) (UniProt - Q9SKR2)	ER-PM tethering and endocytic recycling	No	No	Interacts with TMV movement protein (MP) Pd localization signal (PLS) for cell-to-cell transport and participates in the formation of virus replication sites	Suggested that SYTA relocates to the Pd cavity after TMV infection	[59,60,61,62]
Pd secretory proteins						
*A. thaliana* (1,3)-β-glucanase 1 (AtBG1) (UniProt - Q9M2M0)	Callose-degrading enzymes that positively control Pd permeability	Yes	Asn291	Colocalizes with callose near Pd orifices	No	[36,63,64]
*A. thaliana* (1,3)-β-glucanase 2 (AtBG2) (UniProt P33157)		Yes	No	Colocalizes with callose near Pd orifices in the extracellular space	AtBG2 is not delivered to the extracellular space in TMV-infected cells, but associates with viral MP in Pd cytoplasmic sleeve	[36,64]
*A. thaliana* (1,3)-β-glucanase 3 (AtBG3) (UniProt F4j270)		Yes	No	Constitutive Pd-associated enzyme but not stress-regulated	No	[36,64]
*A. thaliana β-1,3-glucanase_putative Pd-associated protein (AtBG_ppap) (UniProt Q9FHX5)		Yes	No	Localizes to the Pd neck region	No	[35,36,65]
*A. thaliana* Class 1 reversibly glycosylated polypeptide (At^C1^RGP) (UniProt Q9SRT9)	At^C1^RGP acts as a negative Pd regulator. Despite having no signal sequence, it is found in the GA and ultimately in the Pd	No	No		N/A	[66,67,68]
*A. thaliana* Callose synthase (CalS) (UniProt Q9AUE0)	Callose-synthesizing enzyme encoded by glucan synthase-like (GSL) gene that negatively controls Pd permeability	No (needs exocyst-positive organelle (EXPO)-mediated secretion for Pd localization)	No	CalS localizes at callose depositions	No	[36,37,69]
*A. thaliana* formin-like protein 1 (UniProt Q9SE97) and 2 (UniProt O22824) (AtFH1 and AtFH2)	Negatively regulates Pd permeability by interacting with actin filaments	Yes	Multiple Asn sites	Localizes in the Pd cavity and interacts with actin	N/A	[70,71]
*A. thaliana* β-1,6-N-acetylglucosaminyl transferase-like enzyme (GnTL) (UniProt Q9SUZ8)	Interacts with calreticulin and probably serves as a negative regulator of Pd permeability	Yes	Asn287 and Asn316	Colocalizes with callose-binding protein near Pd orifices	N/A	[72]
*A. thaliana Pd callose-binding proteins 1,2 and 3 (PDCB1-3) (At5g61130)	Pd callose-binding protein that negatively controls Pd permeability	Yes	Asn154 and Asn179	Localizes to the Pd neck	No	[73,74]
*A. thaliana* Pd-located protein 1 (PDLP1) (UniProt Q8GXV7)	Membrane receptor-like protein with two extracellular DUF26 domains. PDLP1 overexpression causes restricted cell-to-cell trafficking. Acts as a negative Pd regulator by promoting callose deposition. Stimulates the transport of viruses that use tubule-guided movement by redundantly interacting with tubule-forming MPs within Pds	Yes	No	PDLP1 is targeted to Pd via the Brefeldin A–sensitive secretory pathway and resides at Pd with its C-terminus in the cytoplasmic space and its N-terminus in the apoplast.	No	[75,76,77,78]
*A. thaliana* Pd located protein 5 (PDLP5) (UniProt Q8GUJ2)	A member of the PDLP family that has 30% amino acid sequence identity to PDLP1. Contains sphingolipid binding motif in the TMD. Acts as a negative Pd regulator by promoting callose deposition. Delays systemic movement of TMV	Yes	Asn69 and Asn132	PDLP5 localizes inside the central Pd region similar to TMV MP. However, PDLP5/MP overlap is not complete	Transmembrane secretory protein with ectopic localization	[79,80,81,82]
Tobacco pectin methylesterase (PME) (UniProt Q9LEBO)	Non-direct regulator of Pd permeability that participates in the de-methylesterification of cell wall HG through the formation of methanol	Yes	Asn43, Asn101 and Asn220 in proPME	Immunogold localization of PME is preferentially around Pd	No	[83,84]

^*^ glycosylphosphatidylinositol-anchored protein (GPI-AP) [85].
